# Individualized structural network deviations predict surgical outcome in mesial temporal lobe epilepsy: a multicenter validation study

**DOI:** 10.1097/JS9.0000000000002928

**Published:** 2025-07-02

**Authors:** Li Feng, Honghao Han, Jiajie Mo, Yongwen Huang, Kailing Huang, Chunyao Zhou, Xuyang Wang, Junquan Zhang, Zhiquan Yang, Dingyang Liu, Kai Zhang, Huafu Chen, Qiangqiang Liu, Rong Li

**Affiliations:** a Department of Neurology, Xiangya Hospital, Central South University, Changsha, P.R. China; b The Clinical Hospital of Chengdu Brain Science Institute, School of Life Science and Technology, University of Electronic Science and Technology of China, Chengdu, P.R. China; c MOE Key Laboratory for Neuroinformation, High-Field Magnetic Resonance Brain Imaging Key Laboratory of Sichuan Province, University of Electronic Science and Technology of China, Chengdu, P.R. China; d Department of Neurosurgery, Xiangya Hospital, Central South University, Changsha, P.R. China; e National Clinical Research Center for Geriatric Disorders, Xiangya Hospital, Central South University, Changsha, Hunan, P.R. China; f Department of Neurosurgery, Clinical Neuroscience Center Comprehensive Epilepsy Unit, Ruijin Hospital, Shanghai Jiao Tong University School of Medicine, Shanghai, P.R. China; g Clinical Neuroscience Center, Ruijin Hospital Luwan Branch, Shanghai Jiao Tong University School of Medicine, Shanghai, P.R. China; h Department of Neurosurgery, Tiantan Hospital, Capital Medical University, Beijing, P.R. China

**Keywords:** individualized, machine learning, mesial temporal lobe epilepsy, structural network, surgical outcome

## Abstract

**Background:**

Surgical resection is an effective treatment for medically refractory mesial temporal lobe epilepsy (mTLE), however, more than one-third of patients fail to achieve seizure freedom after surgery. This study aimed to evaluate preoperative individual morphometric network characteristics and develop a machine learning model to predict surgical outcome in mTLE.

**Methods:**

This multicenter, retrospective study included 189 mTLE patients who underwent unilateral temporal lobectomy and 78 normal controls between February 2018 and June 2023. Postoperative seizure outcomes were categorized as seizure-free (SF, *n* = 125) or non-seizure-free (NSF, *n* = 64) at a minimum of 1-year follow-up. The preoperative individualized structural covariance network (iSCN) derived from T1-weighted MRI was constructed for each patient by calculating deviations from the control-based reference distribution, and further divided into the surgery network and the surgically spared network using a standard resection mask by merging each patient’s individual lacuna. Regional features were selected separately from bilateral, ipsilateral, and contralateral iSCN abnormalities to train support vector machine models, validated in two independent external datasets.

**Results:**

NSF patients showed greater iSCN deviations from the normative distribution in the surgically spared network compared to SF patients (*P* = 0.02). These deviations were widely distributed in the contralateral functional modules (*P* < 0.05, false discovery rate corrected). Seizure outcome was optimally predicted by the contralateral iSCN features, with an accuracy of 82% (*P* < 0.05, permutation test) and an area under the receiver operating characteristic curve (AUC) of 0.81, with the default mode and frontoparietal areas contributing most. External validation in two independent cohorts showed accuracy of 80% and 88%, with AUC of 0.80 and 0.82, respectively, emphasizing the generalizability of the model.

**Conclusion:**

This study provides reliable personalized structural biomarkers for predicting surgical outcome in mTLE and has the potential to assist tailored surgical treatment strategies.

## Introduction

Mesial temporal lobe epilepsy (mTLE), the most common form of drug-resistant epilepsy in adults, is characterized by recurrent seizures originating from the sclerotic hippocampus^[1]^. Surgical resection, primarily anterior temporal lobectomy (ATL), has been demonstrated to effectively reduce seizures in patients with mTLE. However, approximately 40% of individuals continue to experience persistent seizures following surgery, and the factors contributing to surgical failure remain not entirely understood^[2]^.

Inadequate surgical resection of the epileptogenic zone has been implicated as a potential contributing factor to surgical failure^[3]^. A growing body of evidence suggests that the epileptogenic regions in mTLE are abnormally organized into epileptogenic networks (ENs) rather than being confined to focal sources^[4]^, highlighting the necessity for improved definition of ENs to optimize surgical strategies. With a shift towards predicting surgical outcomes based on distributed brain networks rather than the traditional epileptogenic zone model, neuroimaging studies utilizing functional MRI (fMRI)^[5]^ and diffusion tensor imaging (DTI)^[6,7]^ _ENREF_14 have demonstrated associations between brain connectivity profiles and postoperative outcomes in TLE patients. While brain network biomarkers derived from fMRI and DTI have received considerable attention in the development of predictive models for seizure outcomes, these modalities are not routinely acquired in the preoperative setting.

Structural T1-weighted (T1w) MRI is a routine preoperative assessment for mTLE patients^[8]^. The structural covariance network (SCN), constructed from T1w images, captures the common covariation in morphological characteristics across different brain areas^[9]^. Previous research has identified regional changes in structural covariance, primarily in orbitofronto-temporal regions^[10]^ and subcortical structures such as the hippocampus and thalamus^[11]^, suggesting potential associations with common epileptic processes in ENs. In contrast to traditional group-level SCN analysis, the construction of an individualized SCN (iSCN)^[12]^ using normative modelling allows for the statistical analysis of each patient’s deviation from the normative pattern. With high accessibility and clinical practicality of T1w MRI scans in preoperative evaluation, iSCN holds translational value in capturing the preoperative individual structural network characteristics and guiding surgical prognosis in mTLE.

Based on the theory of incomplete resection of ENs, we hypothesized that patients who fail to achieve postoperative seizure freedom would show more extensive iSCN deviations in the surgically spared network. In this light, we aimed to evaluate the relationship between preoperative structural network abnormalities and postoperative outcomes, and to develop a predictive model for mTLE patients using the iSCN features and artificial intelligence (AI) methods. In accordance with the Transparency In The reporting of Artificial Intelligence (TITAN) Guidelines 2025 for transparent and responsible AI use in neuroimaging^[13]^, no generative AI was involved; all analyses and interpretations were performed under full human oversight. Finally, we sought to validate the generalizability of the predictive model using two independent, multicenter mTLE datasets.

## Materials and methods

### Participants

This multicentre, retrospective study enrolled 244 patients with mTLE from three local hospitals between February 2019 and August 2022. Patients from Xiangya Hospital, Central South University were used for model training and internal validation. Patients from Ruijin Hospital, Shanghai Jiaotong University School of Medicine and Tiantan Hospital, Capital Medical University were assigned as two independent external validation cohorts (Supplemental Digital Content Figure 1, available at: http://links.lww.com/JS9/E732). The diagnosis of mTLE was established for all patients based on the International League Against Epilepsy classification^[14]^, which involved a comprehensive evaluation including clinical history, seizure description, neurological examination, scalp video-electroencephalography (EEG) recordings, and MRI assessment. The inclusion criteria for patients were as follows: (1) evidence of unilateral temporal lobe seizures confirmed through scalp or intracranial video-EEG recordings; (2) presence of MRI-documented pathology in the epileptogenic mesial temporal lobe, accompanied by hippocampal sclerosis; (3) concordant findings of hypometabolism in the interictal temporal lobe on positron emission tomography imaging; and (4) treatment with ATL or selective amygdalohippocampectomy. Patients presenting with extratemporal, multifocal, or generalized epilepsy, or epilepsy syndrome were excluded from the study. Postoperative outcomes were assessed at the 1-year follow-up using the Engel classification system^[15]^, consistent with international guidelines for epilepsy surgery evaluation^[16]^. This standardized timepoint was selected based on established evidence demonstrating that: (1) the first postoperative year represents the period of highest seizure recurrence risk^[2]^, and (2) 1-year seizure freedom strongly predicts long-term prognosis^[17]^. Patients were classified as SF (Engel class IA) or NSF (Engel class IB-IV) accordingly.

**HIGHLIGHTS**

- We developed a predictive model based solely on routine T1-weighted MRI to forecast individual surgical outcomes in patients with mesial temporal lobe epilepsy (mTLE).
- Surgical outcomes in mTLE were significantly associated with widespread abnormalities in the spared structural covariance network, especially within contralateral functional modules.
- The MRI-based machine learning model achieved 82% accuracy in the primary cohort and demonstrated robust generalizability, with accuracies of 80% and 88% across two independent external cohorts, supporting its potential for personalized outcome prediction and clinical decision-making.

The control group consisted of 78 individuals who were either employees of Xiangya Hospital of the Central South University or members of the local community who responded to a local advertisement. These controls met the health screening requirements and underwent a neurological examination, which produced normal findings and no structural abnormalities on MRI.

All data were de-identified prior to analysis. The study was approved by the Ethics Committee of Xiangya Hospital, Central South University (201912528). All participants gave written informed consent prior to study enrollment. The work has been reported in line with the Standards for the Reporting of Diagnostic Accuracy Studies criteria^[18]^ and complies with the TITAN Guidelines 2025 for the responsible use and transparent reporting of AI in neuroimaging research^[13]^. Specifically, no generative AI was used during data analysis, interpretation, or manuscript preparation. The entire process was conducted under full human oversight and verification.

### MRI acquisition and preprocessing

The primary cohort: the T1w images were obtained utilizing a Siemens Trio 3.0 T scanner at Xiangya Hospital of Central South University. The scanning parameters were as follows: repetition time (TR) = 2110 ms, echo time (TE) = 3.18 ms, flip angle = 9°, field of view (FOV) = 234 × 234 mm, matrix size = 320 × 320, voxel size = 0.73 × 0.73 × 0.73 mm, and slices = 256.

The external validation cohort 1: the T1w images were acquired utilizing a Philips Ingenia 3.0 T scanner at Tiantan Hospital, Capital Medical University, Beijing. The scanning parameters for this cohort were: TR = 2200 ms, TE = 2.26 ms, flip angle = 8°, FOV = 256 × 256 mm, matrix size = 256 × 256 voxel size = 1 × 1 × 1 mm, and slices = 192.

The external validation cohort 2: the T1w images were acquired utilizing a United-Imaging 3.0 T scanner at Ruijin Hospital, Shanghai Jiaotong University School of Medicine. The scanning parameters for this cohort were: TR = 7.5 ms, TE = 3.4 ms, flip angle = 7°, FOV = 220 × 240 mm, matrix size = 439 × 480, voxel size = 0.5 × 0.5 × 0.5 mm, and slices = 360.

To generate grey matter (GM) images with a voxel size of 1.5 × 1.5 × 1.5 mm^3^, we employed the Computational Anatomy Toolbox^[19]^ (CAT12, http://dbm.neuro.uni-jena.de/cat) for T1w structural data processing. Tables 1 and 2 offer a summary of the quality assurance statistics for all preprocessed T1w images. The resulting GM images were then parcellated into 246 cortical and subcortical regions of interest (ROIs) based on the Human Brainnetome Atlas^[20]^. These ROIs were assigned to seven brain modules: frontal, temporal, parietal, insular, limbic, occipital, and subcortical networks.

**Table 1:** Demographic and clinical information in training cohorts

	Xiangya Hospital cohort			
Variable	Controls (*n* = 78)	SF (*n* = 43)	NSF (*n* = 28)	*P*
Age (year)	31.21 ± 9.24	29.41 ± 11.52	30.32 ± 10.04	*P*_1,2_ = 0.351^a^, *P*_2,3_ = 0.736^a^, *P*_1,3_ = 0.668^a^
Gender (female/male)	46/32	19/24	17/11	*P*_1,2_ = 0.118^b^, *P*_2,3_ = 0.174^b^, *P*_1,3_ = 0.872^b^
IQR	87.22 ± 1.89	87.37 ± 0.91	87.16 ± 2.34	*P*_1,2_ = 0.586^a^, *P*_2,3_ = 0.891^a^, *P*_1,3_ = 0.875^a^
Onset age (year)	N/A	16.37 ± 13.00	13.25 ± 9.22	*P*_2,3_ = 0.275^a^
Duration (year)	N/A	13.05 ± 9.89	17.07 ± 8.61	*P*_2,3_ = 0.083^a^
Frequency (per month)	N/A	14.84 ± 19.29	21.38 ± 31.98	*P*_2,3_ = 0.283^a^
Laterality (left/right)	N/A	24/19	15/13	*P*_2,3_ = 0.853^b^
HS	N/A	20 (0.46)	11 (0.39)	*P*_2,3_ = 0.549^b^
FCD	N/A	2 (0.05)	3 (0.11)	*P*_2,3_ = 0.329^b^
HS + FCD	N/A	0 (0)	1 (0.04)	*P*_2,3_ = 0.212^b^
Tumoral	N/A	6 (0.14)	2 (0.07)	*P*_2,3_ = 0.350^b^
Gliosis	N/A	13 (0.30)	11 (0.39)	*P*_2,3_ = 0.223^b^
CCMs	N/A	2 (0.05)	0 (0)	*P*_2,3_ = 0.247^b^
Surgery (ATR/SELAH)	N/A	39/4	27/1	*P*_2,3_ = 0.356^b^

Data are presented as mean ± standard deviation.

ATL, anterior temporal lobectomy; CCMs, cerebral cavernous malformations; FCD, focal cortical dysplasia; HS, hippocampal sclerosis; IQR, image quality assessment; NA, not available; NSF, non-seizure-free; SelAH, selective amygdalohippocampectomy; SF, seizure-free.

^a^ Two-sample *t*-tests.

^b^ Chi-square tests.

**Table 2:** Demographic and clinical information in validation cohorts

	Tiantan Hospital cohort			Ruijin Hospital cohort		
Variable	SF (*n* = 70)	NSF (*n* = 31)	*P*-value	SF (*n* = 12)	NSF (*n* = 5)	*P*-value
Age (year)	28.60 ± 10.10	29.62 ± 10.99	*P* = 0.460^a^	32.67 ± 13.07	30.20 ± 19.36	*P* = 0.761^a^
Gender (female/male)	38/32	13/18	*P* = 0.252^b^	6/6	2/3	*P* = 0.706^b^
IQR	84.99 ± 1.42	85.23 ± 1.24	*P* = 0.319^a^	94.35 ± 0.47	94.82 ± 0.25	*P* = 0.428^a^
Onset age (year)	15.08 ± 8.63	15.70 ± 8.54	*P* = 0.742^a^	13.25 ± 11.23	13.80 ± 10.66	*P* = 0.645^a^
Duration (year)	13.51 ± 9.48	13.91 ± 10.18	*P* = 0.987^a^	19.42 ± 14.54	14.40 ± 11.01	*P* = 0.902^a^
Frequency (per month)	15.21 ± 18.85	19.33 ± 23.61	*P* = 0.401^a^	17.46 ± 21.08	12.00 ± 11.42	*P* = 0.933^a^
Laterality (left/right)	33/37	13/18	*P* = 0.628^b^	7/5	1/4	*P* = 0.149^b^
HS	16 (0.23)	10 (0.32)	*P*_2,3_ = 0.854^b^	8 (0.68)	2 (0.40)	*P*_2,3_ = 0.309^b^
FCD	15 (0.21)	4 (0.13)	*P*_2,3_ = 0.210^b^	1 (0.08)	1 (0.20)	*P*_2,3_ = 0.496^b^
HS + FCD	24 (0.35)	10 (0.32)	*P*_2,3_ = 0.103^b^	1 (0.08)	1 (0.20)	*P*_2,3_ = 0.496^b^
Tumoral	0 (0)	1 (0.03)	*P*_2,3_ = 0.131^b^	1 (0.08)	0 (0)	*P*_2,3_ = 0.506^b^
Gliosis	15 (0.21)	6 (0.19)	*P*_2,3_ = 0.560^b^	1 (0.08)	0 (0)	*P*_2,3_ = 0.110^b^
CCMs	0 (0)	0 (0)	N/A	0 (0)	1 (0.20)	N/A
Surgery (ATR/SELAH)	70/0	31/0	N/A	12/0	5/0	N/A

Data are presented as mean ± standard deviation.

ATL, anterior temporal lobectomy; CCMs, cerebral cavernous malformations; FCD, focal cortical dysplasia; HS, hippocampal sclerosis; IQR, image quality assessment; NA, not available; NSF, non-seizure-free; SelAH, selective amygdalohippocampectomy; SF, seizure-free.

^a^ Two-sample *t*-tests.

^b^ Chi-square tests.

### Presurgery network definition

**Figure 1.**
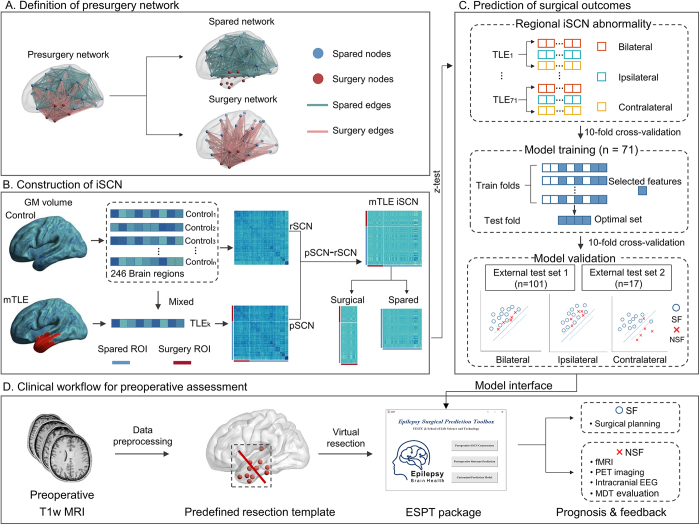
Schematic overview of the analytical process. (A) Preoperative and postoperative T1-weighted MRIs from patients at Xiangya Hospital were utilized to generate a standardized surgical resection mask based on the 246 Human Brainnetome Atlas. This mask further divides the presurgical brain network into the surgery network and the surgically spared network. (B) iSCN is defined as the *Z*-score of the difference between the pSCN and rSCN, representing the degree of deviation from the normative structural covariance. (C) Significantly abnormal structural covariance edges from the rSCN (|*Z*| > 2) were defined for each mTLE patient. Three categories of iSCN features included: (1) bilateral, (2) ipsilateral, and (3) contralateral. Then, SVM models were constructed utilizing three types of optimal feature sets to predict seizure outcomes through CV10. External validation was performed on two cross-site datasets. (D) The proposed method is incorporated into a clinical workflow for preoperative evaluation. TLE patients first underwent preoperative T1-weighted MRI scanning and preprocessing, followed by a virtual resection utilizing a predefined template. Utilizing our ESPT package interfaced with the trained model, prognostic results were generated to guide clinical surgical decision-making. ISCN, individualized structural covariance network; rSCN, reference structural covariance network; pSCN, perturbed structural covariance network; SVM, support vector machine; mTLE, mesial temporal lobe epilepsy; SF, seizure-free; NSF, non-seizure-free.

**Figure 2.**
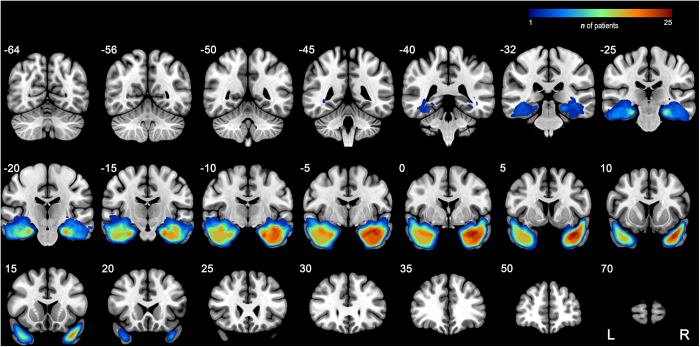
Overlap maps of resection regions in the patient cohort from Xiangya Hospital. The color bar indicates the number of patients.

The process of defining the presurgical network (Fig. 1A) involved identifying surgical resection regions in the 246-Atlas. First, we utilized images from 41 patients at Xiangya Hospital with postoperative T1w MRI to manually draw their individual resection areas in the preoperative T1w MRI space, thus defining the resected tissues (Fig. 2). Then, individual surgical nodes were determined by overlapping each ROI in the 246-Atlas with the individual resection area utilizing a threshold of 50%. For right mTLE patients, the individual surgical nodes were flipped to the left, and all individual surgical nodes were merged to generate a group-level resection mask containing 21 ROIs. Accordingly, the presurgical network was partitioned into two subnetworks: (1) the surgery network, which comprises nodes that underwent surgery and their related edges; and (2) the spared network, which includes nodes that were unaffected by surgery and their corresponding edges.

### iSCN construction

In this study, we employed a Network Template Perturbation method^[21]^ to construct the iSCN (Fig. 1B). First, a reference structural covariance network (rSCN) was established on the control group (*n* subjects) using the partial Pearson’s correlation between each pair of GM volumes across all brain regions (age, sex and total intracranial volume as covariates). Then, a single patient *k* was incorporated into the control group, producing a perturbed SCN (pSCN) in the *n* + 1 individuals (n controls and 1 patient). The iSCN was then directly computed as the difference between pSCN and rSCN (pSCN–rSCN). Finally, in accordance with previous studies^[12]^, the iSCN was transformed into *Z*-score format as follows:

$$z=\frac{iSCN}{\frac{1-rSCN^{2}}{n-1}}$$

Intuitively, networks exhibiting high absolute *Z-*scores (|*Z*|) represent high deviations from normality. Abnormal connections were defined by a threshold of |*Z*| > 2. Each ROI was categorized as ipsilateral or contralateral to the seizure focus, and its regional abnormalities were evaluated at three levels by summing the |*Z*| values of abnormal connections linked to spared nodes bilaterally, ipsilaterally, and contralaterally.

### Predictive modeling for surgical outcome by machine learning

To predict individual surgical outcomes, a machine learning procedure consisting of three layers – feature input, feature selection, and classification – was developed (Fig. 1C). The three levels of regional abnormality of spared nodes (bilateral, ipsilateral, and contralateral) were separately used as input features. Feature selection was performed with the *F*-score method^[22]^, first selecting the top 20 features and incrementally increasing in steps of 5 until optimal classification performance was achieved. Classification was carried out utilizing a support vector machine (SVM) model with a linear kernel, evaluated by a 10-fold cross-validation (CV10) strategy, which measured accuracy, sensitivity, specificity, and AUC. The SVM models produced regional weights, and the average weight of each functional network was computed based on Yeo’s 7-network^[23]^ parcellation. To assess the generalizability of the predictive model, external validation was conducted on 101 mTLE patients from Tiantan Hospital and 17 mTLE patients from Ruijin Hospital.

### Statistical analysis

The global abnormalities of the surgery and spared networks were measured by summing all the abnormal connections in each respective network. Modular abnormalities were measured by summing the regional abnormalities in each module, which were further categorized into bilateral, ipsilateral, and contralateral categories. Two-sample *t*-tests with Benjamini–Hochberg false discovery rate (FDR) correction for multiple comparisons (*P* < 0.05) were employed to determine statistically significant differences in global and modular abnormalities between the SF and NSF cohorts. The statistical significance of the classifier was evaluated utilizing a permutation test with 5000 iterations and a significance threshold of *P* < 0.05.

## Results

### Characteristics of the study cohort

Of the 244 mTLE patients initially screened, 55 were excluded due to loss to follow-up (*n* = 37) or lack of preoperative MRI data (*n* = 18), leaving 189 patients for the final analysis. Of these, 125 patients achieved SF and 64 patients were classified as NSF. The patient cohorts were further classified into six etiological subtypes based on MRI findings and histopathological evaluations: (1) hippocampal sclerosis (HS) (*n* = 67), (2) focal cortical dysplasia (FCD) (*n* = 26), (3) HS combined with FCD (HS + FCD) (*n* = 37), (4) tumor (*n* = 10), (5) gliosis (*n* = 46), and (6) cerebral cavernous malformations (CCMs) (n = 3). A patient flowchart is illustrated in Supplemental Digital Content Figure 1, available at: http://links.lww.com/JS9/E732. The main demographic and clinical characteristics are summarized in Tables 1 and 2.

### iSCN abnormalities in subnetworks

**Figure 3.**
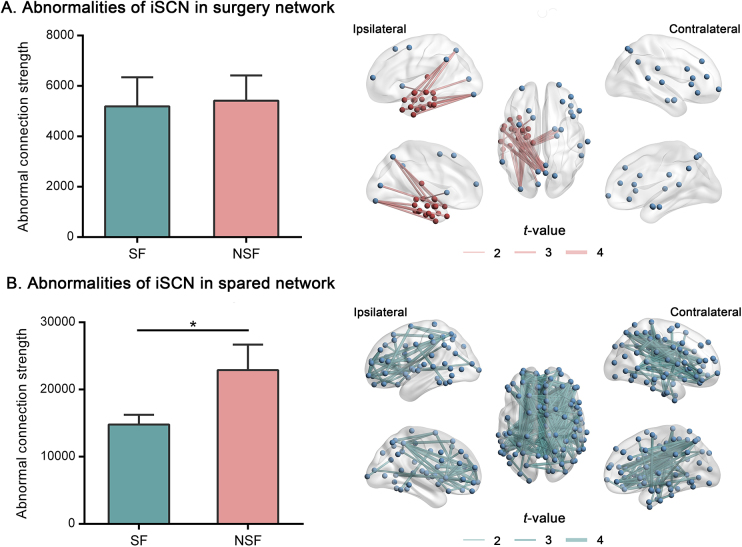
Comparisons of iSCN abnormal connection strength between SF and NSF patients in the surgery network (A) and the spared network (B). The histograms (left panel) illustrate group differences in iSCN abnormality strength. The brain maps (right panel) depict the results of the two-sample *t*-tests conducted in the surgery network and spared network, and the top 1% of edges with the highest *t*-values were plotted. Edge thickness corresponds to *t*-values. *P < 0.05. SF, seizure-free; NSF, non-seizure-free.

Statistical analysis showed no significant difference in the strength of iSCN abnormality between the SF and NSF groups in the surgery network (*P* > 0.05; Fig. 3A, left). In contrast, the spared network exhibited significantly greater abnormality strength in NSF patients compared to SF patients (*P* < 0.05, uncorrected; Fig. 3B, left). To visualize the aberrant iSCN patterns in both subnetworks, we selected the top 1% of edges with the highest *t*-values (Fig. 3, right). This indicates that NSF patients displayed more extensive iSCN abnormalities in the spared network relative to the surgery network. Given the established association between etiology and surgical outcomes in mTLE^[24,25]^, we assessed the relationship between etiology and preoperative iSCN abnormalities. One-way analysis of variance performed within each center revealed no significant differences among the six etiological subtypes (*P* > 0.05, Supplemental Digital Content Figure 2, available at: http://links.lww.com/JS9/E732), likely due to limited sample sizes. Based on prior reports indicating that HS, tumor, and CCMs are more commonly associated with SF^[26,27]^, whereas FCD, HS + FCD, and gliosis are linked to NSF^[28–30]^, we reclassified the etiologies into two broader categories. Subsequent comparisons of preoperative iSCN abnormalities between these two groups revealed significant differences in Tiantan Hospital and Ruijin Hospital (*P* < 0.05), but not in Xiangya Hospital (*P* > 0.05) (Supplemental Digital Content Figure 3, available at: http://links.lww.com/JS9/E732).

**Figure 4.**
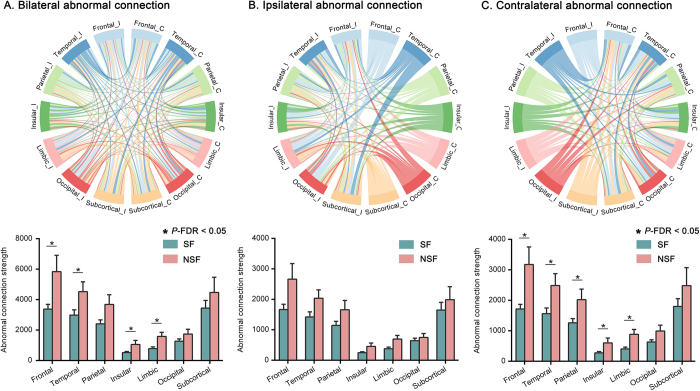
Comparisons of iSCN abnormal connection strength in bilateral (A), ipsilateral (B), and contralateral (C) functional brain modules in the surgically spared network. The upper chord diagrams represent brain connections between each pair of brain modules. Edge thickness reflects the differences in iSCN abnormality between SF and NSF patients. The lower histograms illustrate the differences in each functional brain module. *P < 0.05, FDR corrected, the *P* value was obtained by two-sample *t*-test. C, contralateral; I, ipsilateral; SF, seizure-free; NSF, non-seizure-free; FDR, Benjamini–Hochberg false discovery rate.

Upon partitioning the spared network into seven modules, we observed widespread differences in the strength of iSCN abnormality across multiple functional modules. Specifically, NSF patients demonstrated significantly higher abnormality strength in the frontal, temporal, insular, and limbic regions compared to SF patients (*P* < 0.05, FDR corrected, Fig. 4A). In addition, we categorized the modular abnormality strength into ipsilateral and contralateral components. No significant differences were detected between the SF and NSF patients in any of the ipsilateral modules (all *P* > 0.05, FDR corrected, Fig. 4B); whereas, contralateral modules exhibited significantly greater abnormality strength in NSF patients compared to SF patients in the frontal, temporal, parietal, insular, and limbic regions (*P* < 0.05, FDR corrected, Fig. 4C).

### Predictors of surgical outcome

**Figure 5.**
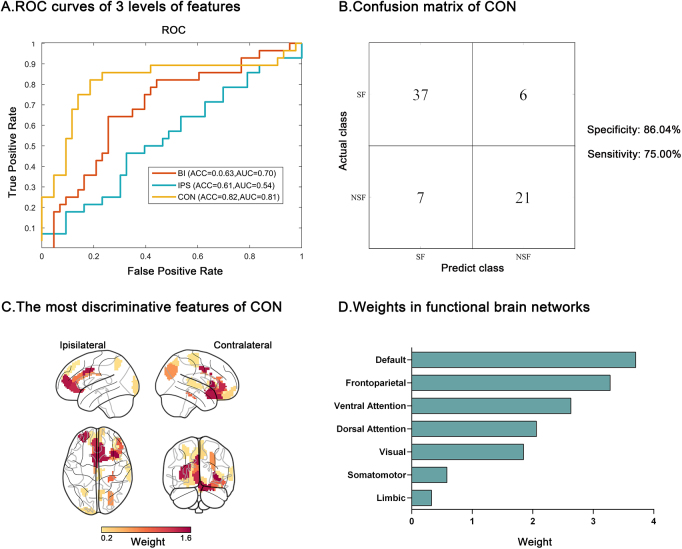
Prediction performance of SVM models utilizing three levels of abnormality strength for each node as features. (A) ROC curves. The contralateral model exhibits the best prediction performance with an accuracy of 82% (*P* < 0.05, permutation test) and an AUC of 0.81. (B) Sensitivity and specificity were estimated at 75% (21/28) and 86% (37/43), respectively. (C) The most discriminative features (top 20 weight) for the contralateral model classification are primarily situated in the insula, cingulate gyrus, orbital and middle frontal areas, parahippocampal gyrus, and precuneus. (D) The DMN is the primary contributor to prediction based on the weight of statistical features in Yeo_7 network parcellation. SVM, support vector machine; ROC, receiver operating characteristic; AUC, area under the curve; DMN, default mode network; BI, bilateral, IPS, ipsilateral, CON, contralateral; SF, seizure-free; NSF, non-seizure-free.

Figure 5 depicts the classification performance based on the top 25 ROI abnormalities with the highest *F*-scores, selected as classification features across the three levels. The contralateral features demonstrated the strongest ability to discriminate between the SF and NSF groups, achieving an accuracy of 82% (*P* < 0.05, permutation test) and an AUC of 0.81. Supplemental Digital Content Table 1, available at: http://links.lww.com/JS9/E732 presents the model’s performance for each of the ten cross-validation folds. In contrast, the ipsilateral features exhibited poor performance, with an accuracy of only 61% and an AUC of 0.54 (*P* = 0.16, permutation test). The bilateral features also performed poorly, attaining an accuracy of 63% and an AUC of 0.70 (*P* < 0.05, permutation test). Consistent results were observed when varying the number of features (e.g. top 20, top 30, Supplemental Digital Content Table 2, available at: http://links.lww.com/JS9/E732), suggesting a significant correlation between contralateral abnormal connections and surgical outcomes in mTLE patients.

To interpret the model, we selected and visualized the top 20 weights as the most discriminative features across the CV10, based on an empirical value from previous research^[31]^ (Fig. 5C, Supplemental Digital Content Tables 3–5, available at: http://links.lww.com/JS9/E732). To further verify the stability of the selected features, we visualized features that appeared in at least 90% of the cross-validation iterations^[32]^ (Supplemental Digital Content Figure 4, available at: http://links.lww.com/JS9/E732). Both model interpretation strategies consistently highlighted the significant importance of the limbic system, temporal regions, and frontoparietal cortices. Moreover, we evaluated the feature importance of seven functional modules and discovered that the primary contributors to classification were primarily concentrated in the default mode network (DMN) (top 1) and the frontoparietal network (FPN) (Fig. 5D).

**Figure 6.**
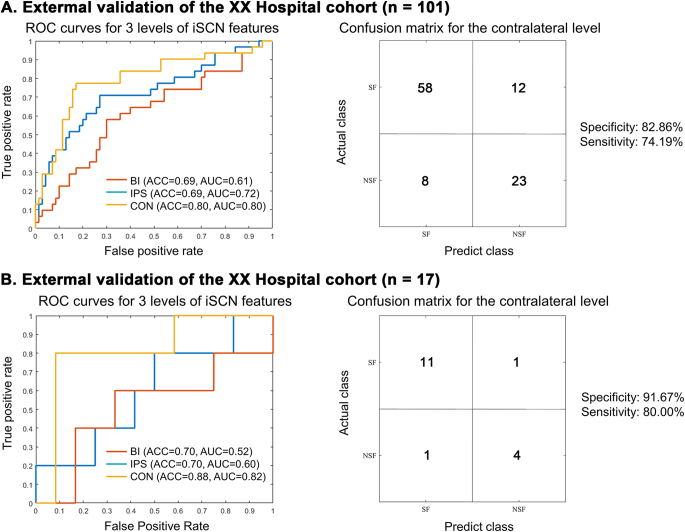
Model performance in external validation. (A) External validation results from Tiantan Hospital based on the trained model, employing three levels of abnormality strength for each node as features. The contralateral model also demonstrates the best prediction performance with an accuracy of 80% and an AUC of 0.80 (left panel). Specificity and Sensitivity were estimated at 82.86% (58/70) and 74.19% (23/31), respectively (right panel). (B) External validation results from Ruijin Hospital based on the trained model, employing three levels of abnormality strength for each node as features. The contralateral model also demonstrates the best prediction performance with an accuracy of 88% and an AUC of 0.82 (left panel). Sensitivity and specificity were estimated at 80% (4/5) and 91.67% (11/12), respectively (right panel). ROC, receiver operating characteristic; AUC, area under the curve; BI, bilateral, IPS, ipsilateral, CON, contralateral; SF, seizure-free; NSF, non-seizure-free.

We applied the trained model to two independent external datasets from different centers. The external validation results of Tiantan Hospital cohort were also optimal when utilizing contralateral features, yielding an accuracy of 80% and an AUC of 0.80. The classification performance was suboptimal when employing either ipsilateral or bilateral features, with both achieving an accuracy of 69% and AUCs of 0.72 and 0.61, respectively (Fig. 6A). For Ruijin Hospital cohort, the external validation results were also optimal when utilizing contralateral features, yielding an accuracy of 88% and an AUC of 0.82. The classification performance was suboptimal when employing either ipsilateral or bilateral features, with both achieving an accuracy of 70% and AUCs of 0.60 and 0.52, respectively (Fig. 6B). Additionally, we evaluated the accuracy of surgical outcome prediction across different etiological subtypes and centers. The model maintained robust predictive performance across various etiologies and across all three participating centers (Supplemental Digital Content Table 6, available at: http://links.lww.com/JS9/E732), further supporting its clinical applicability.

## Discussion

This study aimed to investigate the preoperative individualized structural covariance architecture in mTLE patients and to develop an optimal machine learning model for predicting surgical outcomes. By incorporating virtual resection information, we classified the preoperative iSCN into two subnetworks. Specifically, we discovered that NSF patients exhibited more significant individualized structural covariance shifts compared to SF patients in the surgically spared network. These abnormalities were widely distributed across the contralateral frontal, temporal, parietal, insular, and limbic modules. Employing an SVM with CV10, we found that the contralateral iSCN abnormality in the surgically spared network could predict surgical outcomes with an accuracy of 82% and an AUC of 0.81, with the DMN and FPN contributing most. In addition, external validation results from two independent cross-site datasets, which separately achieved an accuracy of 80% with an AUC of 0.80, and an accuracy of 88% with an AUC of 0.82, demonstrated the generalizability of our approach. Our findings emphasize the predictive value of preoperative structural covariance deviations in the contralateral retention network for surgical outcomes and provide a potential personalized brain biomarker that can be leveraged for clinical surgical evaluation in mTLE.

Surgical resection remains the most prevalent treatment approach for mTLE; however, a proportion of patients do not achieve postoperative seizure freedom. Previous studies utilizing a plethora of neuroimaging methods based on functional and structural connectivity have suggested that preoperative neuroimaging could serve as a biomarker for predicting epilepsy surgery outcomes^[5,33]^. However, the majority of these analyses neglected the crucial role of the resection zone in brain network analysis, leading to bias in the accurate prediction of surgical outcomes in drug-resistant epilepsy^[34]^. In this study, we optimized the prediction model by incorporating the surgical resection mask into the preoperative iSCN analyses and prediction models. We discovered that, in comparison to controls, patients with poor surgical outcomes exhibited greater shifts in the structural covariance pattern, particularly in the contralateral surgically spared network, than those who achieved seizure freedom. Prior research has indicated that individuals with epilepsy whose intracranial EEG studies indicated complex propagation patterns, potentially involving contralateral distant networks, tended to have less favorable surgical outcomes in terms of achieving seizure freedom after surgery^[35]^. Moreover, several neuroimaging studies have also demonstrated anatomical^[36]^, functional^[5]^, and metabolic^[37]^ changes in the contralateral hemisphere in patients with TLE and have evaluated the relationship between these changes and surgical outcomes, supporting the hypothesis of contralateral involvement as a potential pathophysiological mechanism. Our findings are in line with these previous observations and highlight the prognostic value of individualized contralateral structural covariance architecture in mTLE surgery.

We further found that iSCN shifts in the surgically spared network were extensively distributed across the frontal, temporal, parietal, insular, and limbic functional modules, particularly in the contralateral brain regions. This distribution of our structural network findings aligns with growing evidence from multimodal neuroimaging studies. DTI studies have consistently indicated that white matter abnormalities in these regions predict poorer outcomes, with increased nodal abnormalities correlating with reduced SF rates^[38]^. Similarly, resting-state fMRI reveals that lower network integration, especially in the contralateral temporoinsular cortex, associated with post-surgical seizure persistence^[5]^. Furthermore, high preoperative modular structural-functional coupling in the DMN has emerged as a reliable predictor of seizure recurrence after surgery^[39]^. This multimodal convergence strongly supports the biological validity of our structural network approach and its ability to capture key features of EN. Considering that epilepsy is a systemic disorder affecting large-scale networks^[40]^, epileptiform discharges originating from a focal brain area can propagate to distant contralateral regions through the corpus callosum and midline structures^[41,42]^, with the limbic system and frontoparietal cortices being frequently affected by mTLE^[43]^. Therefore, the observed abnormal structural covariance of the GM in the temporo-limbic circuit and the frontoparietal modules may be indicative of the underlying epileptogenic processes occurring between these brain regions. The extensive fiber connections linking the temporal and limbic cortices with the frontoparietal cortices may offer the anatomical foundation for these abnormalities^[44,45]^. Moreover, the NSF patients showed more abnormal connections in the surgically spared network, suggesting that in addition to the common epileptogenic focus, other EN abnormalities in mTLE necessitate further attention, as they may potentially inform the development of individualized resection strategies.

Machine learning offers promising tools for establishing the most appropriate surgical predictive model based on multimodal data utilizing various algorithms^[46,47]^. The success of the model hinges on its ability to generalize effectively. In this study, we implemented two crucial safeguards. First, all feature selection and model construction took place in a CV10 procedure. Second, we evaluated the model generalizability utilizing two completely independent, untouched validation samples, a process known as external validation. We extracted three levels of iSCN features from the surgically spared network for each patient, including the bilateral, ipsilateral, and contralateral abnormal connection strengths. Employing the SVM classifier, we found that the contralateral network model exhibited the best predictive performance, achieving an accuracy of 82% and an AUC of 0.81 in the internal dataset. The external validation analysis further demonstrated that the contralateral network model most effectively predicted outcomes in the Tiantan Hospital cohort, with an accuracy of 80% with an AUC of 0.80 and in the Ruijin Hospital cohort, with an accuracy of 88% with an AUC of 0.82. The observed performance variation between validation cohorts may be attributed to differences in patient characteristics, MRI acquisition parameters, or surgical practices across centers. Although the substantial sample size disparity (101 vs. 17 patients) could introduce statistical variance, the model maintained clinically robust accuracy in both settings, demonstrating its stability across heterogeneous clinical environments.

Our work contributes to the growing evidence supporting the utility of individualized morphological networks in the preoperative evaluation of TLE patients, particularly as a prognostic tool for predicting seizure outcomes. Moreover, our results indicated that the brain nodes contributing most significantly to the predictive model were primarily situated in the DMN and frontoparietal association areas. This aligns with previous research indicating that DMN hub regions exhibit abnormalities in TLE across various modalities, such as GM volume^[48]^, structural^[49]^, and functional connectivity^[50]^. The DMN is a widely distributed set of brain regions involving the limbic system, mesial and lateral temporal cortex, precuneus, and medial prefrontal areas, which is selectively impaired during seizures with loss of consciousness^[51]^. Regarding surgical outcomes, our group has previously demonstrated increased temporal variation of functional connectivity in the DMN in NSF patients^[52]^. Here, we further establish that individualized structural covariance deviations of the DMN are the most informative features for predicting surgical outcomes and can be incorporated into routine clinical practice to enhance surgical outcome prediction.

Considering the robust performance of our developed model and the accessibility of T1w MRI, we have integrated and released our model in the publicly available neuroimaging package, referred as the Epilepsy Surgical Prediction Toolbox (ESPT), to facilitate its incorporation into clinical workflows (Fig. 1D). The process begins with mTLE patients undergoing preoperative T1w MRI scanning and preprocessing. Then, a virtual resection is performed utilizing a predefined template developed in this study. Finally, our ESPT package generates a prognostic result to guide clinical surgical decision-making for a given patient. This open-source software has the potential to advance neuroimaging data analysis and aid in the surgical evaluation of epilepsy.

Nevertheless, it is important to acknowledge that this study has several limitations. First, we defined a group-level resection mask from a subset of patients for whom postoperative T1w MRI data were available. However, in actual surgical procedures, resection regions vary from patient to patient. Theoretically, creating an individualized virtual resection mask for each patient would allow a more accurate definition of the surgically spared network and could potentially improve predictive performance. Second, while our model was validated in two independent cohorts, the sample size of the second external validation cohort (*n* = 17) remains limited, which may affect the generalizability of the results. This constraint arises from the necessity of including only patients with ≥1 year of postoperative follow-up data, which is a rigorous criterion that ensures clinical relevance but complicates rapid recruitment. Despite this, future larger multicenter sample sizes would be beneficial to optimize the model and verify its use as a potential tool for preoperative clinical evaluation. Third, although our T1-weighted imaging approach offers superior clinical accessibility, the multimodal integration could enhance network characterization. Future prospective studies will collect multimodal imaging data in our extended cohort to systematically evaluate how integration might enhance predictive accuracy. Fourth, as a non-randomized study, our findings may be influenced by selection biases inherent to clinical decision-making. Future prospective randomized studies are needed to mitigate these biases and further validate the predictive model. Finally, our study utilized the standardized 1-year follow-up interval that aligns with current surgical outcome assessment guidelines^[15,16]^, this time frame may not fully capture late postoperative seizure recurrences. The performance characteristics of our predictive model should be further validated in extended follow-up studies to confirm its durability for long-term prognosis assessment.

## Conclusions

In summary, our study demonstrates that the unique preoperative iSCN characteristics, extracted from T1w MRI, serve as valuable predictors of surgical outcomes in patients with refractory mTLE. Our findings indicate that patients exhibiting extensive SCN aberrations in the contralateral surgically spared network are less likely to achieve seizure freedom after surgery. In particular, the structural covariance deviations in the DMN and frontoparietal association regions are highly predictive of seizure outcomes. Our research sheds light on the key components of ENs in mTLE and advances the clinical application of personalized neuroimaging biomarkers as prognostic tools for surgical outcomes.

## Data Availability

The data generated in this study are available upon request from the corresponding author. All source code and trained models are publicly available at https://github.com/RongLiNeuroLab/Surgical-prediction.

## Research registration unique identifying number (UIN)

Researchregistry11042.
